# Clinicopathological risk factors for recurrence after neoadjuvant chemotherapy and radical hysterectomy in cervical cancer

**DOI:** 10.1186/1477-7819-11-301

**Published:** 2013-11-25

**Authors:** Huali Wang, Lin Zhu, Weihua Lu, Hui Xu, Yunhai Yu, Yongxia Yang

**Affiliations:** 1 Gynecology Department, The Second Hospital of Shandong University, No. 247 Beiyuan Street, Jinan, Shandong 250033, China; 2 Paediatrics Department, The First Hospital of Ningyang County, Taian, Shandong 271400, China

**Keywords:** Cervical cancer, Radical hysterectomy, Recurrence, Risk factors

## Abstract

**Background:**

Cervical cancer is one of the common gynecological malignancies with a high recurrence rate after surgery. This study aimed to analyze the clinicopathological risk factors for recurrence after the surgical treatment of cervical cancer and provide the basis for the prevention of recurrence and an improvement of prognosis.

**Methods:**

A total of 424 cervical cancer cases between 1 January 1998 and 31 December 2011 undergoing surgical treatment were studied retrospectively, of which 23 cases had recurrences. Relevant recurrence risk factors were evaluated by univariate and multivariate analyses between recurrence group and non-recurrence group.

**Results:**

Using univariate analysis, tumor differentiation, clinical stage, pelvic lymph node metastasis, postoperative radiotherapy and postoperative chemotherapy were related to recurrence of cervical cancer. Multivariate COX model analysis revealed that pelvic lymph node metastasis and postoperative chemotherapy had an impact on recurrence rate. Moderately and highly differentiated tumor, advanced clinical stage, and positive pelvic lymph nodes indicated a high recurrence rate of cervical cancer. Postoperative chemotherapy and radiotherapy can effectively reduce the recurrence rate.

**Conclusions:**

In conclusion, cervical lymph node metastasis and postoperative chemotherapy are two independent factors for recurrence of cervical cancer after radical surgery.

## Background

Cervical cancer is one of the common gynecological malignancies, with nearly 500,000 women developing the disease each year worldwide [1]. Cervical cancer staging generally runs from stage I, which is non-invasive, to stage IV, in which the carcinoma has extended beyond the true pelvis or has involved (biopsy proven) the mucosa of the bladder or rectum, based on the International Federation of Gynecology and Obstetrics (FIGO) stage system [2]. Most women with early-stage tumors can be cured by radical surgery. However, the local tumor recurrence rate after radical surgery is up to 30% [3], and recurrence has become the main reason for poor prognosis and a decreased survival rate.

Recently, though the application of surgery and adjuvant therapy, early cervical cancer patients have a high survival rate, especially for type Ib and IIa patients, with the 5-year survival rate up to about 75 to 90% [4]. Those with a large cervical lesion or positive pelvic lymph nodes are usually treated with a combination of radiotherapy with concomitant chemotherapy. In the last two decades, several studies have shown that neoadjuvant chemotherapy followed by radical hysterectomy is able to obtain very satisfactory results in locally advanced cervical cancer [5]. However, not much is known about whether neoadjuvant chemotherapy, including preoperative and postoperative therapy, can improve the recurrence rate of cervical cancer. In addition, cervical cancer recurrence can be affected by many factors, such as clinical stage, histological type, histological grade, treatment method, self-status, and others [6,7]. Evaluating these factors and looking for an effective treatment to improve the recurrence rate has become a hot topic for gynecologic oncology. In this study, we aimed to find out the clinical and pathological risk factors for cervical cancer recurrence through retrospective analysis of 424 cervical cancer patients undergoing radical hysterectomy.

## Methods

### Patients

A total of 424 women admitted to The Second Hospital of Shandong University from 1 January 1998 to 31 December 2011 were included in this study. According to the FIGO classification, there were 127 cases with stage IA, 97 cases with stage IB (15 with stage IB1 and 82 with stage IB2), 112 cases with stage IIA and 88 cases with stage IIB cancer. This study was approved by the Ethics Committee of The Second Hospital of Shandong University and formal informed consent was obtained from the patients.

### Preoperative treatment

The patients with stage IB2, IIA or IIB carcinoma were subjected to neoadjuvant therapy since their tumor diameters were larger than 4 cm and good outcomes may not have been available by direct surgical treatment. Finally, 139 of 282 cases were treated with neoadjuvant chemotherapy or radiotherapy preoperatively (Table 1).

**Table 1:** Number of patients treated with different approaches

	**Different approaches**	**Number**
Preoperative treatment	Neoadjuvant chemotherapy	88
	Radiotherapy	51
	None	285
Surgical treatment	Abdominal surgery	328
	Laparoscopic surgery	96
Postoperative treatment	Chemotherapy	182
	Radiotherapy	179
	None	63

The following neoadjuvant chemotherapy schemes were used: i) didecyl phthalate (DDP) 20 mg/m^2^ (first 5 days) + 5-fluorouracil (5-FU) 0.75 g/m^2^ (first 5 days); ii) DDP 50 mg/m^2^ (first day) + bleomycin (BLM) 25 mg/m^2^ (first 3 days) + vincristine (VCR) 1 mg/m^2^ (first day) + mitomycin (MMC) 10 mg/m^2^ (first day); and iii) DDP 50 mg/m^2^ (first day) + BLM 25 mg/m^2^ (first 3 days) + VCR 1 mg/m^2^ (first day). There were 18, 13 and 57 patients received the first, second and third scheme of the neoadjuvant chemotherapy, respectively. A cobalt 60 machine (AECL, Canada) or 8 MeV medical linear accelerators (siemens, Germany) were used for radiation therapy. The whole pelvis was irradiated based on the anteroposterior view with an area of 13 to 15 cm × 15 to 18 cm. The radiation treatment was performed five times a week with a dosage of 1.8 to 2 Gy. The dose was then increased to 25 to 30 Gy, two to three times a week, with the bladder and rectum blocked by a 3 to 4 cm × 13 to 15 cm lead plate. Iridium-192 intracavitary brachytherapy combined with pelvic external radiation was then applied; the radiation dose of iridium-192 intracavitary brachytherapy was 6 to 7 Gy each time with a total dose of 30 to 42 Gy every week. The preoperative radiotherapy was finished within 6 to 8 weeks.

### Surgical treatment

All the patients were treated by radical hysterectomy and pelvic lymphadenectomy. Among them, 328 cases were treated with abdominal surgery and the other 96 cases with laparoscopic surgery (Table 1).

### Postoperative treatment

Patients with deep muscular involvement, vascular invasion or a tumor diameter larger than 4 cm received two to four cycles of chemotherapy (DDP + 5-FU) for 7 to 10 days after surgery, while patients with two of above conditions were treated with radiotherapy. If a positive lymph node, positive surgical margin or paracervical involvement occurred, radiotherapy was also considered. A total of 182 of 246 cases underwent chemotherapy and 179 of 256 cases received whole pelvic external radiation within 1 month (Table 1).

The numbers of patients treated with the different approaches are summarized in Table 1.

### Follow-up

All cases were followed up by letter and telephone. The follow-up time varied from a minimum of 2 months to a maximum of 165 months.

### Statistical analysis

SPSS 13.0 software (SPSS Inc., Chicago, IL, USA) was used for statistical analysis. Eight clinicopathological factors that may be related to the recurrence of cervical cancer were used for analysis. The recurrence time was calculated from the day of surgery to the last day of follow-up on a monthly basis. Patients who died from other diseases were normalized as a loss to follow-up. Recurrence rates were compared by the log-rank test. *P* < 0.05 was used to indicate statistical significance. The factors found to have a significant impact on postoperative recurrence of cervical cancer based on univariate analysis were entered into a Cox proportional hazards regression model for multivariate analysis.

## Results

### Clinical data of postoperative recurrence

All 424 patients enrolled in this study experienced surgical treatment, and recurrence occurred in 23 cases. The median recurrence time was 15.54 months (range, 12.5 to 48 months).

### Univariate analysis of clinicopathological factors for recurrence

Univariate risk analysis was performed first (Table 2). Data showed that tumor histological grade (*P* < 0.001) and clinical stage (*P* < 0.001) were well correlated with cervical cancer recurrence after surgery. Patients with moderately and highly differentiated cancer or ≤ IB stage cancer were subject to lower recurrence rates when compared to patients with poorly differentiated cancer or > IB stage cancer, respectively. Furthermore, the recurrence rate was significantly higher in patients with positive pelvic lymph nodes (16.038%) than in patients with negative pelvic lymph nodes (1.887%, *P* < 0.001). Tumor recurrence occurred more frequently in patients without postoperative chemotherapy and radiotherapy than in patients with postoperative chemotherapy (*P* = 0.007) or radiotherapy (*P* = 0.000), but there was no significant difference in recurrence between patients with preoperative adjuvant therapy and those without preoperative adjuvant therapy (*P* = 0.597). The recurrence rate was not associated with the pathological type of cancer (that is, no significant difference existed between patients with squamous carcinoma and patients with adenocarcinoma or others (*P* = 0.275)). The age of the patient also did not affect the recurrence rate (*P* = 0.179).

**Table 2:** Univariate analysis of clinicopathological factors for cervical cancer recurrence after surgery

**Factor**	**Total number (n)**	**Recurrence number (n)**	**Recurrence rate (%)**	**Log-rank test (*P *** **value)**
Age (years)				
≤ 40	57	1	1.754	0.179
> 40	367	22	5.995	
Pathological type				
Squamous carcinoma	328	20	6.098	0.275
Adenocarcinoma and others	96	3	3.125	
Histological grade				
Poorly differentiated	246	21	8.537	0.000
Moderately or highly differentiated	178	2	1.124	
Clinical stage				
≤ IB	224	2	0.893	0.000
> IB	200	21	10.500	
Adjuvant therapy				
With	139	8	5.755	0.597
Without	143	10	6.993	
Pelvic lymph node				
Positive	106	17	16.038	0.000
Negative	318	6	1.887	
Postoperative radiotherapy				
With	179	8	4.469	0.000
Without	77	8	15.584	
Postoperative chemotherapy				
With	182	5	2.747	0.007
Without	64	7	10.938	

### Multivariate analysis of clinicopathological factors for recurrence

All the single risk factors affecting postoperative recurrence rate were further studied using COX proportional hazards regression model. Histological grade, clinical stage, postoperative radiotherapy, postoperative chemotherapy and postoperatively confirmed positive lymph node were included in this model to find out which risk factors were vital for recurrence by forward stepwise regression on the level of α = 0.05. The results (Table 3) revealed that postoperatively confirmed positive lymph nodes (degree of risk, 14.649; 95% CI 1.781 to 120.520; *P* = 0.013) and postoperative chemotherapy (degree of risk, 0.252; 95% CI 0.074 to 0.856; *P* = 0.027) were significant independent predictors of cervical cancer recurrence.

**Table 3:** Multivariate analysis of clinicopathological factors for cervical cancer recurrence after surgery

**Factor**	**Model parameters**	**Standard error**	**Parametric test statistic**	**Degree of freedom**	***P *** **value**	**Degree of risk**	**95% CI**	
							**Upper limit**	**Lower limit**
Lymph node (positive or negative)	2.684	1.075	6.233	1	0.013	14.649	1.781	120.520
Postoperative radiotherapy	−0.717	0.597	1.440	1	0.230	0.488	0.151	1.575
Postoperative chemotherapy	−1.379	0.624	4.884	1	0.027	0.252	0.074	0.856
Histological grade	−0.913	0.790	1.335	1	0.248	0.401	0.085	1.889
Clinical stage	0.265	0.842	0.099	1	0.753	1.303	0.250	6.787

## Discussion

Radical hysterectomy with lymphadenectomy is the routine treatment for early (FIGO IB-IIA) invasive cervical cancer, and patients with early cervical cancer can be cured. However, long-term prognosis is poor due to the high rate of recurrence. Many factors have been reported to have an impact on cervical cancer recurrence. In the current study, we evaluated the clinicopathological factors in 424 patients with stage IB to IIA cervical cancer who underwent surgical treatment and found that the clinical stage, histological grade, pelvic lymph node, postoperative radiotherapy and postoperative chemotherapy were significantly related to recurrence. Furthermore, pelvic lymph node metastasis and postoperative chemotherapy were two independent risk factors for recurrence based on multivariate analysis.

Lymph node metastasis is recognized as an unfavorable factor of cancer recurrence [8,9]. Positive lymph node also indicates poor prognosis in cervical cancer in that the survival rates for patients with lymph node metastases are significantly lower than those for patients with no detectable nodal metastases [10,11]. Girardi and colleagues [12] revealed that the recurrence rate was higher when the parametrial nodes were positive than when they were negative. Similarly, univariate and multivariate analyses in our study showed that the recurrence rate with lymph node metastasis was 14.69 times more than that without lymph node metastasis. Therefore, lymph node metastasis has potential prognostic value and systematic lymphadenectomy plays a vital role in the treatment for node-positive patients.

In addition to lymph node metastasis, the clinical stage and tumor differentiation degree were also demonstrated to be closely associated with cervical recurrence [13,14]. Our data showed a worse prognosis in lower differentiated cervical cancer patients compared with well-differentiated cervical cancer patients, with recurrence rates of 8.537% and 1.124%, respectively. Patients with lower stage cancer (≤ IB stage) were subject to lower recurrence rates when compared to patients with advanced cancer (> IB stage).

There are different opinions as to whether neoadjuvant therapy, especially neoadjuvant chemotherapy, can prolong patient survival time and reduce the relapse rate. Some clinical studies have shown that neoadjuvant chemotherapy can further reduce the tumor volume, improve the outcome of surgical treatment, and eventually give a better prognosis. Chen and colleagues [15] reported that, compared to the surgery group alone, 3-year and 5-year survival rates of interventional neoadjuvant chemotherapy combined with surgery are significantly improved (*P* < 0.05). However, some other researchers deny the conclusion that neoadjuvant chemotherapy can reduce recurrence and improve long-term survival [16]. Our results showed that the recurrence rate was not significantly different between patients with and without preoperative adjuvant therapy.

Currently, a majority of studies show that postoperative radiotherapy and chemotherapy have a good effect on preventing recurrence and improving prognosis in cervical cancer patients, especially for patients with pelvic lymph node metastases. Kukura and colleagues [17] confirmed the importance of whole pelvic radiotherapy by analysis of 72 stage IB cervical cancer patients. Yamakawa and colleagues [18] presented a significantly decreased recurrence rate with postoperative radiotherapy plus chemotherapy compared to those without adjuvant treatment. Liu and colleagues [19] showed that there was a higher survival rate in patients with stage IB and IIA carcinoma of the cervix who received adjuvant chemoradiotherapy after radical hysterectomy than those undergoing radical hysterectomy alone. Rotman and colleagues [20] confirmed that pelvic radiotherapy after radical surgery can significantly reduce the risk of recurrence and prolong progression-free survival in women with stage IB cervical cancer. In our study, 179 of 256 patients received postoperative radiotherapy and had a recurrence rate of 4.469%, whereas the recurrence rate in those without radiotherapy was 15.584%. There was a significant difference between these two groups (*P* = 0.000). Among 246 patients that needed further postoperative chemotherapy, 182 patients with postoperative chemotherapy had a recurrence rate of 2.747% while 64 patients without chemotherapy had a recurrence rate of 10.93% (*P* = 0.007). Besides the univariate analysis, postoperative chemotherapy displayed a potential prognostic value for cervical cancer recurrence through the multivariate analysis.

In the multivariate COX proportional hazards regression model analysis, clinical stage, histological grade and postoperative radiotherapy did not show a statistically significant difference, though each factor alone showed statistical difference in the single factor analysis. The reason may be that the effect of each factor in single factor analysis could be overlapped by the combined effects of several other factors in COX multivariate analysis, or that its role could be replaced by other risk factors. However, in spite of very few clinical reports showing agreement, our data showed postoperative chemotherapy alone is an independent factor for the recurrence of cervical cancer. This result is likely due to the long follow-up time, with the role of chemotherapy manifesting gradually with the extension of follow-up. Of course, larger sample and multicenter prospective studies are needed to validate this result.

## Conclusion

Based on the findings of this study, we conclude that a positive lymph node is an important indicator for recurrence of cervical cancer after radical surgery. Surgeons should pay attention to lymph node resection in the surgical treatment of node-positive patients. At the same time, we advocate the application of postoperative chemotherapy for special patients, which is beneficial to reduce the recurrence of cervical cancer.

## Abbreviations

5-FU: 5-fluorouracil; BLM: Bleomycin; DDP: Didecyl phthalate; FIGO: International federation of gynecology and obstetrics; VCR: Vincristine.

## Competing interests

The authors declare that they have no competing interests.

## Authors’ contributions

HW wrote the first draft of this report. LZ and WL performed the operation. HW, LZ, WL, HX, YY, and YY performed the postoperative management. HX supervised the writing of the paper. HW is the guarantor of the paper. All authors have contributed significantly, and all authors are in agreement with the content of the manuscript. All authors read and approved the final manuscript.
